# Controllable Ion-Repulsion Enables Rapid and Trace-Level Detection of Hydrophobic Sulfonylurea Herbicides with ICPMS/MS without the Organic Mode

**DOI:** 10.1021/acs.analchem.5c01663

**Published:** 2025-06-13

**Authors:** Bassam Lajin, Walter Goessler

**Affiliations:** † Institute of Chemistry, ChromICP, University of Graz, Universitaetsplatz 1, 8010 Graz, Austria; ‡ Institute of Chemistry, Analytical Chemistry for the Health and Environment, 27267 University of Graz, Universitaetsplatz 1, 8010 Graz, Austria

## Abstract

The inductively coupled plasma tandem mass spectrometry (ICPMS/MS) is emerging as a detector for compounds tagged with a nonmetal, but can be hampered by poor compatibility with organic eluents. Herein, we describe an alternative approach to using high fractions of organic solvents for the detection of hydrophobic compounds while presenting a novel sulfur speciation application involving the determination of a common and widely used class of hydrophobic sulfonylurea herbicides. The approach is based on controllable retention using an ion-repulsion concept, and it is shown that this approach enables minimizing the amount of organic eluent needed for speciation analysis, eliminating the need for the organic ICPMS mode and therefore achieving uncompromised sensitivity. The applied approach enabled fast analysis within 8 min with a mobile phase containing as little as 10% methanol. The limit of detection was 0.3 μg S L^–1^ (injection volume 20 μL), which is on par with the lowest achievable LOD for sulfur speciation with ICPMS/MS detection, highlighting the utility of the ion-repulsion approach in overcoming the current limitations of the organic ICPMS mode commonly used with ICPMS detection. Spiking experiments showed the developed method to be directly applicable to a river water matrix with no need for sample preparation or internal standards unlike currently used methods based on ESIMS/MS detection. The ion-repulsion concept is generally applicable to speciation analysis with ICPMS detection for ionizable analytes, provides similar control over retention to organic solvents, and aids in avoiding the limitations of the organic ICPMS mode.

## Graphical abstract

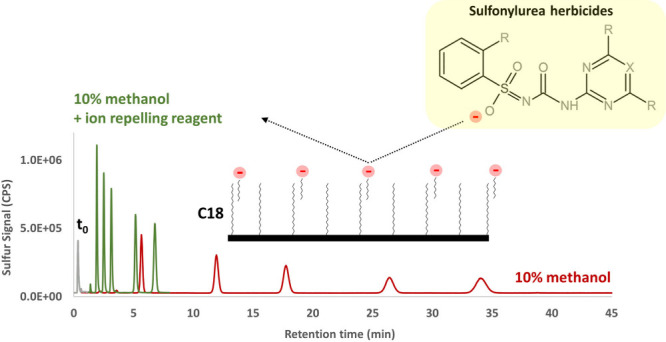

## Introduction

The inductively coupled plasma tandem mass spectrometry ICPMS is an element-selective technique that is gaining increasing popularity as a chromatographic detector due to its advantages relative to other commonly employed techniques. In particular, ICPMS is less prone to matrix suppression effects than commonly used chromatographic detectors such as electrospray ionization mass spectrometry (ESIMS). This eliminates the need for isotopically labeled internal standards for each and every analyte, which is ideally required with HPLC-ESIMS but can be limited by the availability and high cost. Moreover, the lower susceptibility of ICPMS to matrix effects when used as a chromatographic detector when compared to other techniques such as ESIMS enables direct injection of samples without the need for extensive sample cleanup. A further major advantage of ICPMS is the element-dependent and usually molecule-independent signal response, which enables accurate quantification in targeted analysis even in the absence of pure standards provided that the atomic composition of the analyte is known. However, the most striking advantage of ICPMS involves nontargeted analysis where simultaneous coupling of ICPMS and high-resolution molecular mass spectrometry can be a powerful tool for the discovery of novel natural and environmentally relevant compounds tagged with a heteroatom due to significant simplification of molecular metabolomic data by acquiring element-selective profiles. This nontargeted analysis approach with ICPMS detection played a major role in the advancement of our knowledge of the occurrence and metabolism of elements such as arsenic^1–3^ and selenium^4,5^ in biological and environmental systems.

Similar breakthroughs to those achieved in arsenic and selenium research involving other major elements of interest such as sulfur, phosphorus, and the halogens have been hampered by polyatomic interferences that prevent the detection of these elements at low levels. The relatively recent advent of tandem mass spectrometry to ICPMS (i.e., ICPMS/MS) enabled addressing this limitation and extended the trace-level detection capability of ICPMS to the nonmetal elements.^6–8^ Although this has opened wide areas in speciation analysis with HPLC-ICPMS/MS, there remains one major limitation preventing the technique from achieving its full potential, namely, the poor compatibility with reversed-phase chromatography, which is by far the most commonly practiced form of liquid chromatography. This incompatibility stems from the poor tolerability of the inductively coupled plasma to high carbon content originating from organic chromatographic eluents, which results in carbon build-up, instrumental drift, and plasma shutdown at concentrations >5–20% v/v depending on the solvent and mobile phase flow rate. To address this issue, significant changes in instrumental setup and conditions, collectively known as the “organic ICPMS mode”, are commonly applied. The most clearly detrimental components of this practice include mobile phase flow splitting by up to 1 + 9 and postcolumn dilution of the column effluent, which lead to significant loss in sensitivity and high limits of detection that can eclipse the advantages of the introduction of tandem mass spectrometry to ICPMS. Depending on the organic content of the mobile phase, flow-splitting may be avoided by using oxygen as a makeup gas. However, the use of oxygen as a makeup gas requires optimization and requires replacing the standard Ni/Cu cones with the more expensive Pt cones to avoid corrosion. Furthermore, a gradient would not be applicable as the oxygen flow rate has to be changed dynamically to accommodate the organic content which is not applicable under current instrumental set-ups. It is also noteworthy that using oxygen may prevent carbon build-up by converting carbon into CO_2_ and prevent plasma-shutdown, but this practice does not eliminate signal suppression resulting from the elevated carbon content in the plasma. We previously showed that an increase in CO_2_ concentration in the plasma results in sharp decrease in sensitivity for elements with high ionization potential such as chlorine^9^

Therefore, the organic ICPMS mode can result in a loss in detectability. Multiple cases demonstrating this loss of sensitivity for hydrophobic compounds are observed in the literature. A typical example involves the study of arsenolipids which has been conducted over many years using the organic ICPMS mode with limits of detection consistently reported within a high concentration range of 1.0–10 μg As L^–1^.^10–12^ These limits of detection are >100-fold higher than those normally achievable in arsenic speciation analysis involving hydrophilic compounds (e.g., dimethylarsinic acid and arsenobetaine) not requiring the ICPMS organic mode (0.005–0.03 μg L^–1^).^13–15^ This has likely resulted in hindering the discovery of novel low abundance arsenolipids such as biosynthesis intermediates that could possibly improve our understanding of the origins of this class of compounds in nature which has been the subject of debate over more than three decades. Another example involves chlorine speciation analysis where an instrumental limit of quantification of 50 μg Cl L^–1^ was observed for the hydrophobic active pharmaceutical ingredient diclofenac (LogP 4.7), which is 50-fold higher than that achievable for perchlorate (1.0 μg L^–1^) under standard experimental setup without sample preconcentration.^9^ There is therefore a pressing need for new chromatographic approaches that help address the observed bottleneck in the detectability of ICPMS when coupled with reversed-phase liquid chromatography.

The presence of nonmetal heteroatoms, particularly sulfur and halogens, significantly increases hydrophobicity and retention in reversed-phase liquid chromatography. Indeed, a review of the literature reveals the limited number of sulfur speciation analysis studies with HPLC-ICPMS/MS and the majority of studies employed <20% v/v organic eluents in order to avoid the organic ICPMS mode and therefore targeted compounds of low hydrophobicity.^16–18^ The achieved limits of detection in these studies were generally within the range of 1.0–20 μg S L^–1^.^16–18^ We found no application for the sulfur speciation analysis of hydrophobic sulfur compounds with HPLC-ICPMS/MS.

The sulfonylurea herbicides belong to a class of increasingly popular compounds of environmental concern with >25 members in this class. These herbicides are more hydrophobic (LogP up to 3) than other commonly used classes such as the phosphonic acids and require >30% v/v organic fraction to enable rapid elution according to previous chromatographic methods,^19,20^ which is not compatible with ICPMS. The sulfonylurea herbicides are among the most commonly employed worldwide^21^ and some of the most commonly used compounds in this class such as nicosulfuron, metsulfuron, chlorsulfuron, tribenuron methyl, and sulfosulfuron have been previously detected in surface and groundwater samples.^22,23^ Although the sulfonylurea herbicides have relatively low toxicity to mammalian health, a major environmental concern of their use involves their phytotoxicity and the their effects on the growth of plant species away from their application area due to their mobility in soil.^24,25^

Ionic amphiphilic reagents have been employed for decades to improve the retention of charged compounds on the hydrophobic C18 stationary phases in reversed-phase liquid chromatography. These reagents are referred to by chromatographers using many terms, the most common of which is “ion-pairing reagents”. This term can be misleading, as it overdominantly reflects a single mechanism of their action. Multiple mechanisms were previously suggested to explain retention in ion-pair chromatography.^26–28^ However, strong evidence has been presented in support for the formation of a primary and secondary ion layers (i.e., an electric double layer) due to the adsorption of the charged “ion-pairing reagent” on the stationary phase and the resulting attraction of counterion ions in mobile phase.^26,27^ This suggests that retention in ion-pair chromatography is more complex than simple formation of neutral ion-pairs or dynamic ion-exchange.^26^ However, a clear implication of this mechanism is that the use of ion-pairing reagents results in not only increased retention of oppositely charged analytes but also decreased retention of similarly charged analytes.^26,27^ Although decreased retention of analytes with a similar charge to the ion-pairing reagent has been experimentally observed since the early days of the technique,^26,27,29^ the ion-repulsion aspect is often overlooked in ion-pair chromatography and has rarely been exploited in practice to address chromatographic challenges.

In previous work, we described the ion-repulsion effects exerted by short-chain (C2–C4) fluorinated carboxylic acids, a commonly used group of ion-pairing reagents, on the retention of hydrophilic compounds and highlighted its possible applications, particularly its potential in speciation analysis of hydrophobic compounds for overcoming the limited compatibility of ICPMS with organic eluents by eluting compounds through controllable electrostatic repulsion rather than merely solvation.^30^ However, the applicability of this approach for hydrophobic compounds and its implementation in a real-world matrix to support its practical utility in speciation analysis with HPLC-ICPMS are yet to be described.

Herein, we present a novel application of HPLC-ICPMS/MS involving the determination of widely used hydrophobic sulfonylurea herbicides in spiked river water matrix, demonstrating that the “ion-repulsion chromatography” concept can be effectively exploited to minimize the organic mobile phase content for the elution of ionizable analytes in order to avoid the application of the organic ICPMS mode and enable fast determination under standard conditions that maintain the lowest limit of detection achievable by the technique which would not be achievable with the organic ICPMS mode.

## Materials and Methods

### Sample Collection and Handling

River water samples (*n* = 4) were collected from the Mur River passing through the city of Graz (location 47°04′30.7″N 15°26′02.4″E) in September 2023 using polypropylene 300 mL Corning collection bottles (Corning, NY, USA). The samples were stored at 4 °C for 24–48 h until analysis. To test the applicability of the method in this matrix, samples were spiked at various concentrations of the sulfonylurea herbicides within the range of 1.0–100 μg S L^–1^ and used for method validation. No sample preparation was employed other than filtration through 0.2-μm Nylon syringe filters (Chromafil Xtra PA-20/13, Macherey-Nagel GmbH, Dueren, Germany) before transfer to 0.7 mL polypropylene HPLC vials (Bruckner, Linz, Austria) for analysis.

### Reagent Preparation

Mobile phase reagents (perfluoroheptanoic acid ≥ 97%, HPLC grade methanol, acetic acid, and ammonia) were purchased from (Sigma-Aldrich, Steinheim, Germany). Purified water (18.2 MΩ·cm) was obtained from a Milli-Q water purification system (Millipore GmbH, Vienna, Austria) and was used for the preparation of standards and the HPLC mobile phases.

Five of the most commonly used sulfonylurea herbicides were selected, namely, nicosulfuron, metsulfuron, chlorsulfuron, tribenuron-methyl, and sulfosulfuron (Sigma-Aldrich, Steinheim, Germany, purity ≥ 95%). The chemical structures of the studied analytes are illustrated in Figure 1.

**Figure 1:**
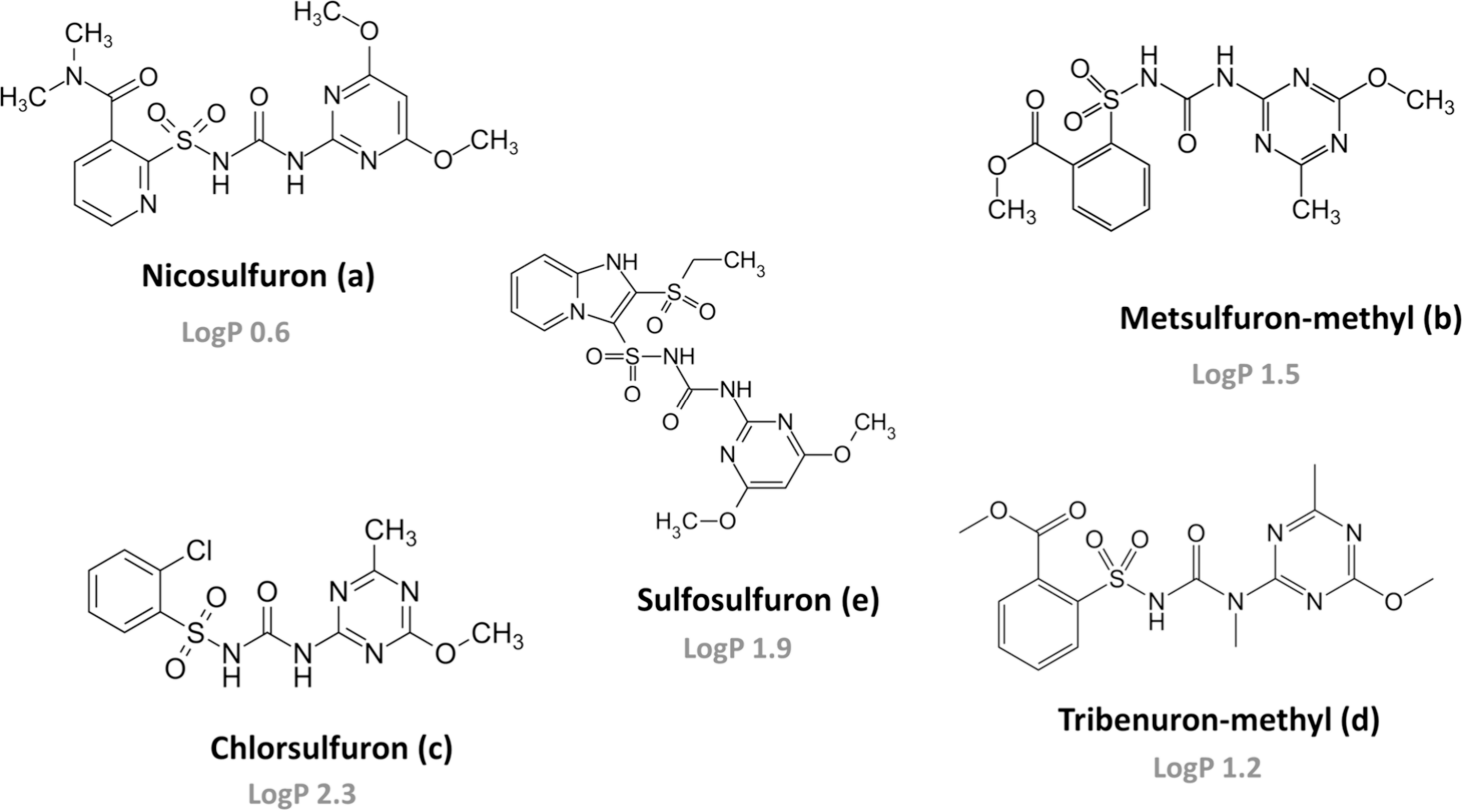
Chemical structure of the target sulfonylurea herbicides.

The class of sulfonylurea herbicides includes at least 25 compounds with Log *P* values generally within the range of 0.2–3.1. Attention was made to select compounds within a representative range of hydrophobicity and Log *P* values (0.6–2.3, shown in Figure 1) in order to enable testing the proposed chromatographic approach under different properties of compounds in this class.

Stock solutions of sulfonylurea herbicide standards were prepared by weighing 10 mg (±0.1) and dissolving in 10 mL of dimethylformamide (≥99%). The resulting 1.0 g L^–1^ solutions were then diluted using the mobile phase buffer (see below) to concentrations within the range of 1.0–1000 μg S L^–1^ to be used for calibration and spiking experiments.

### Instrumental Analysis

For chromatographic separation, an Agilent 1100 system (Agilent Technologies, Waldbronn, Germany) was employed, equipped with an autosampler (ALS G1367C), a quaternary pump (G1311A), a degasser (G1379A), and a column temperature-controlled compartment (G1316A). Separation on a reversed-phase column (YMC Triart-C18, 50 × 2.1 mm, 1.9 μm particle size) was performed isocratically with a mobile phase containing 10% v/v methanol, 0.5 mmol L^–1^ perfluoroheptanoic acid as the “ion-repelling reagent”, and 17 mmol L^–1^ acetic acid with pH adjusted with ammonia to 9.0. All mobile phase reagents were acquired from Sigma-Aldrich, Steinheim, Germany, with purity ≥ 99%). The column was held at a temperature of 40 °C; the mobile phase flow rate was 0.25 mL min^–1^, and the injection volume was 20 μL.

Inductively coupled plasma tandem mass spectrometry (ICPMS/MS) was used as an element-selective detector (Agilent 8900 ICPQQQ, Agilent Technologies, Waldbronn, Germany). A PEEK capillary tubing (ca. 40 cm in length and 0.127 mm I.D.) was used to connect the chromatographic column with the AriMist PEEK nebulizer of the ICPMS/MS system, which was further equipped with a glass Scott double pass spray chamber, Ni/Cu sampler, and skimmer cones and a quartz plasma torch with an inner diameter of 2.5 mm. The detection of sulfur was carried out using oxygen as a reaction cell gas at a flow rate of 0.3 mL min^–1^ in order to produce the mass shift 32 → 48 which distinguishes ^32^S^+^ from the ^16^O^16^O^+^ polyatomic interference. Key parameters were as follows: RF power: 1550 W; RF matching: 1.7; sampling depth: 5.0 mm; nebulizer gas flow rate: 0.65 L min^–1^ and makeup gas flow rate: 0.35 L min^–1^.

## Results and Discussion

We previously provided a systematic investigation of the ion-repulsion effects for short chain (C2–C4) fluorinated carboxylic acids and highlighted the potential of this approach in improving the compatibility issue between reversed-phase liquid chromatography and ICPMS detection.^30^ In the present work, perfluoroheptanoic acid, a longer chain highly hydrophobic member of this series (LogP 4.3), was tested as it provides sufficiently strong adsorption to compete with the hydrophobic analytes (Log *P* 0.6–2.3). Figure 2 illustrates the effect of incorporating perfluoroheptanoic acid as “ion-repelling reagent”. A percentage of 10% v/v methanol in the mobile phase was observed to be the maximum practical methanol concentration employable under standard experimental setup in this work (plasma shut-down occurs at ca. 25% v/v methanol and carbon-build up and instrumental drift are observed at concentrations >15% v/v). It can be seen in Figure 2A that in the absence of perfluoroheptanoic acid significantly higher retention factors (up to *k* 87 for latest eluting peak) than desirable in liquid chromatography for rapid elution (*k* < 20) are observed. Incorporating as little as 0.5 mmol L^–1^ perfluoroheptanoic acid under identical conditions resulted in fast elution and 3–5 fold sharper peaks with proportionately higher S/N ratios (Figure 2B), therefore achieving a desirable chromatographic outcome without having to resort to the organic ICPMS mode involving flow splitting and postcolumn dilution, which would obviously result in significant loss in sensitivity.

**Figure 2:**
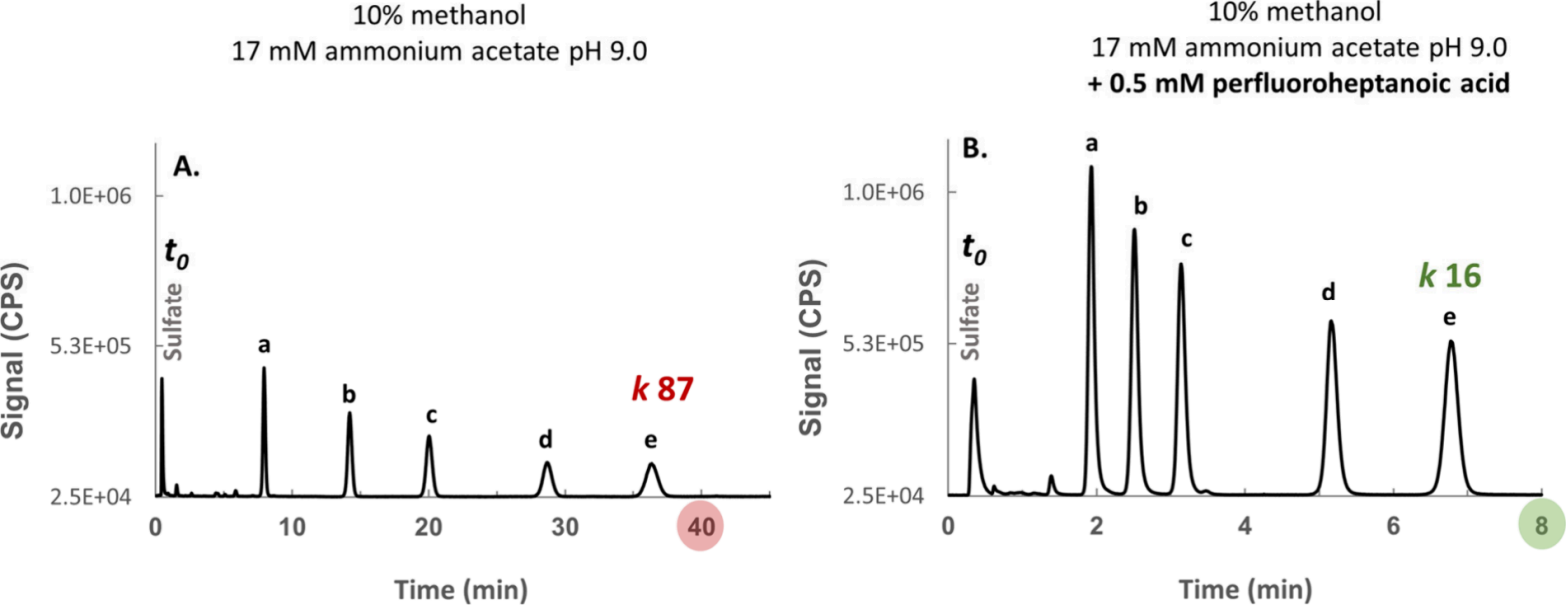
Effects of perfluoroheptanoic acid on the elution of the studied sulfonylurea herbicides from the reversed-phase column, prepared in a standard mixture (100 μg S L^–1^ each). The chromatograms show the elution with a mobile phase containing 10% methanol without perfluoroheptanoic acid (A) and the elution with a mobile phase containing 10% methanol and 0.5 mmol L^–1^ perfluoroheptanoic acid (B). Both mobile phases contain ca. 17 mmol L^–1^ acetic acid with pH adjusted to 9.0 with ammonia. The retention factors were calculated based on the equation *k* = (*t*_r_ – *t*_0_)/*t*_0_, where *t*_r_ is the retention time (min) and *t*_0_ is the void time (estimated at 0.4 min, column dimensions 50 mm × 2.1 mm, flow rate 0.25 mL/min). Note that the sulfonylurea group has a p*K*_a_ within the range of 5.0–6.0 and is fully deprotonated at pH 9.0 conferring the analytes a negative charge subject to ion-repulsion. Faster elution due to ion repulsion (B) resulted in a 3–5-fold reduction in peak width and proportionally higher S/N. (a) Nicosulfuron; (b) Metsulfuron; (c) Chlorsulfuron; (d) Tribenuron methyl; (e) Sulfosulfuron. Sulfate is unretained on reversed-phase columns and is shown for reference. Its retention time might be shortened by ion-exclusion effects, but can roughly represent the void time.

For the separation of ionized organic acids, “ion-repelling reagents” can be dealt with similarly to organic solvents, where a higher elution strength can be achieved by increasing the concentration of the reagent and/or switching to a more hydrophobic member of the series. Increasing the concentration of perfluoroheptanoic acid was found to result in stronger elution, and the logarithmic relationship between the concentration of perfluoroheptanoic acid and the retention factor for the studied analytes was linear (Supporting Information, Figure S1). This enables controlling chromatographic retention in a fashion similar to modifying the fraction of an organic eluent in HPLC-ICPMS separations. A shift in chromatographic selectivity was observed for the early eluting peaks of nicosulfuron and metsulfuron (see the intersection of lines a and b, Supporting Information, Figure S1), highlighting that ion-repulsion chromatography is applicable not only as a general elution approach when using ICPMS detection, but also as a tool to modify separation selectivity.

It is worth noting that although perfluoroheptanoic acid can form micelles, the concentration employed (0.5 mmol L^–1^) is well below the critical micellar concentration (CMC) previously reported in the literature (>30 mmol L^–1^ ^31^). Even though the chromatographic conditions employed in terms of temperature and salt concentration might not be representative of standard conditions used for experimental CMC calculation,^31^ the employed mild conditions (e.g., column temperature 40 °C and 0.02 M salt concentration) would not be expected to result in the massive decrease in CMC required to form micelles at the employed low concentration in this work given the general trends previously reported.^32,33^ Indeed, the observed linear and monophasic relationship between the concentration of perfluoroheptanoic acid and retention factor (Supporting Information, Figure S1) supports the absence of an elution mechanism involving micelle formation within the tested concentration range (0.1–5.0 mmol L^–1^) since micelle formation would result in a biphasic relationship due to enhanced elution at the point of formation of micelles when CMC is exceeded. This further supports the idea that ion-repulsion is the sole major mechanism behind the observed expedited elution achieved by using perfluoroheptanoic acid in the present work.

Elution in ion-repulsion chromatography would be expected to respond to salt concentration opposite to ion-pair chromatography. Indeed, investigating the effects of salt concentration revealed that retention is increased (i.e., restored) with increasing salt concentration, which is clearly attributed to suppression of the underlying electrostatic interactions (Supporting Information, Figure S2). However, concentrations of added ammonium acetate above 70 mmol L^–1^ were observed to lead to decreased retention (Supporting Information, Figure S2). This may be explained by the “salting-out effect”, which would result in a larger influence on the adsorption of the more hydrophobic (Log *P* 4.3) perfluoroheptanoic acid on the stationary phase than that of the sulfonylurea herbicides (Log P 0.6–2.3), resulting in more potent occupation of the C18 phase by the ion-repelling reagent and therefore a decrease in the hydrophobicity of the stationary phase by surface coating with the hydrophilic carboxylate groups. Even though elevated ionic strength can significantly decrease CMC, we believe that micelle formation at the tested salt concentration range is unlikely to explain the decrease in retention observed beyond 70 mmol L^–1^ (Figure S2), as the employed concentration of perfluoroheptanoic acid (0.5 mmol L^–1^) appears to be too distant from the CMC values reported in the literature (>30 mmol L^–1^)^34^ and >0.5 M salt concentration is usually required for >10-fold decrease in CMC of ionic surfactants in general.^33^ However, further experimental work to confirm this may be required.

None of the investigated herbicides were detected in the Mur river water in the present study. However, spiking experiments demonstrated a low limit of quantification of 1.0 μg S L^–1^ (calculated based on the S/N 10 definition) for the detection of the sulfonylurea herbicides in a real-world matrix using the proposed method (Figure 3), which is on par with those previously reported for hydrophilic sulfur-containing compounds with ICPMS/MS detection (0.3–0.5 μg S L^–1^).^16,35^ This limit of quantification is also comparable to that achievable by the commonly employed LC-ESIMS/MS.^36^ However, ESI-based mass spectrometry is known to be prone to severe matrix effects,^37,38^ which usually requires including an isotopically labeled internal standard, ideally for each and every analyte and usually requires sample cleanup. This is a major disadvantage of ESI-based detection compared with the less matrix-prone ICPMS, which generally does not require an internal standard for speciation analysis and allows direct injection without sample purification, as shown in the present work. The method repeatability and accuracy were validated by spiking at two concentration levels (Table 1) where recoveries were found generally within ±20% at the limit of quantification level of 1.0 μg S L^–1^ (Table 1). Calibration was linear within the range of 1.0 μg S L^–1^ – 1.0 mg S L^– 1^ (*r* ^2^ = 0.9999; higher concentrations were not tested).

**Table 1:** Recovery and Repeatability for the Determination of Sulfonylurea Herbicides in the Mur River Water by HPLC-ICPMS/MS^a^

	L1 (1.0 μg L^–1^)	L2 (100 μg L^–1^)
analyte	measured concn (μg S L^–1^)	measured concn (μg S L^–1^)
Nicosulfuron	0.90 ± 0.05	92 ± 3
Metsulfuron	1.0 ± 0.1	96 ± 2
Chlorsulfuron	1.3 ± 0.1	102 ± 4
Tribenuron-methyl	0.80 ± 0.09	98 ± 2
Sulfosulfuron	1.2 ± 0.2	101 ± 3

a The table shows the concentrations (mean ± standard deviation *n* = 3) for the target analytes after spiking with 1.0 μg S L^–1^ (L1) and 100 μg S L^–1^ (L2). None of the target analytes was detected before spiking (LOD 0.3 μg L^–1^).

**Figure 3:**
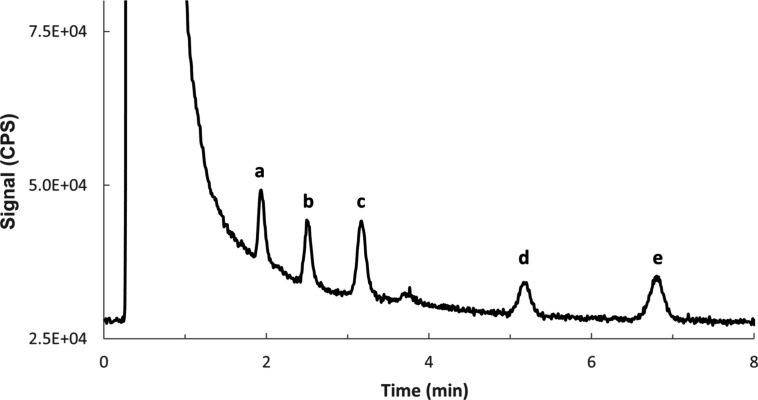
Detection of the sulfonylurea herbicides in spiked Mur river at 1.0 μg S L^–1^ (which is estimated as the limit of quantification based on S/N 10 method). The front peak is attributed to sulfate, which is natively present at ca. 16 mg S L^–1^ in Mur river water. (a) Nicosulfuron; (b) Metsulfuron: (c) Chlorsulfuron; (d) Tribenuron methyl; (e) Sulfosulfuron.

A few considerations of the employed ion-repulsion approach are noteworthy. First, with increasing hydrophobicity of the ion-repelling reagent, the column equilibrium time increases. The column equilibrium time for short chain perfluorinated acids (up to C5) was found to be <20 column volumes. However, perfluoroheptanoic acid (C7) was found to require 50–100 column volumes for complete equilibration (retention time RSD < 0.5%). Furthermore, the sulfonylurea herbicides are a class of moderately hydrophobic compounds (Log *P* < 3). The use of more hydrophobic ion-repelling reagents in combination with small concentrations of stronger eluents than methanol (within the tolerability limits of the plasma) would be expected to extend the applicability of this approach to more hydrophobic classes of compounds (i.e., Log *P* > 3) than the sulfonylurea herbicides, but this requires future testing. Finally, since ion-repulsion chromatography is based on electrostatic interactions, the approach is clearly only applicable to ionizable compounds, and the pH of the mobile phase must be selected to ensure a net charge similar to that of the ion-repelling reagent. For this reason, the mobile phase, pH 9.0, in the present work was set above the p*K*_a_ of the sulfonylurea herbicides (5–6). For positively charged compounds, the fluoroalkylamines^39^ can be used as ion-repelling reagents in combination with a low pH mobile phase. Finally, the developed method can be applicable to other matrices than the investigated river water matrix in the present work, such as tap and groundwater. However, applying the method to matrices with complex sulfur metabolomes (e.g., sulfur-rich plants) may require reoptimizing chromatographic separation. Although the element-selective capability of ICPMS is a unique advantage in nontargeted analysis and novel compound discovery, it can be viewed as a disadvantage when compared to more selective detectors such as ESIMS/MS in targeted analysis of matrices with highly complex elemental metabolomes.

## Conclusion

Ion-repulsion chromatography was shown to serve as a useful approach to enable the elution of the hydrophobic herbicides with minimal organic eluent proportion, eliminating the need for employing a special instrumental setup while achieving a limit of detection on par with the lowest reported for the technique for sulfur speciation by avoiding the use of the organic ICPMS mode. The proposed new method for sulfonylurea herbicides determination can be used as an alternative to current methods based on HPLC-ESIMS/MS, eliminating the need for sample preparation and isotopically labeled internal standards while achieving similar detection limits and offering simultaneous nontargeted sulfur-selective screening possibilities.

## Supplementary Material



## Notes

Influence of perfluoroheptanoic acid (Figure S1) and salt concentration (Figure S2) on retention (PDF)
