# Supplementary material for: Controllable Ion-Repulsion Enables Rapid and Trace-Level Detection of Hydrophobic Sulfonylurea Herbicides with ICPMS/MS without the Organic Mode
Source: Anal Chem. 2025 Jun 13;97(25):13361–7. doi: 10.1021/acs.analchem.5c01663 (PMC12224153; doi:10.1021/acs.analchem.5c01663)
Supplement: Supplementary file 1 [file ac5c01663_si_001.pdf]

Supporting Information

Sulfur speciation analysis of hydrophobic sulfonylurea herbicides with ICPMS/MS detection enabled by controllable ion-repulsion without the organic ICPMS mode

Bassam Lajin* [1, 2] **Walter Goessler** [2]

[1]Institute of Chemistry, ChromICP, University of Graz, Universitaetsplatz 1, 8010 Graz, Austria

[2]Institute of Chemistry, Analytical Chemistry for the Health and Environment, University of Graz, Universitaetsplatz 1, 8010 Graz, Austria

*Correspondence to: bassam.lajin@uni-graz.at

Table of contents

Fig. S1 Influence of perfluoroheptanoic acid on the retention of the studied compounds (page S2)

Fig. S2 Influence of salt concentration on the retention of the studied compounds (page S3)

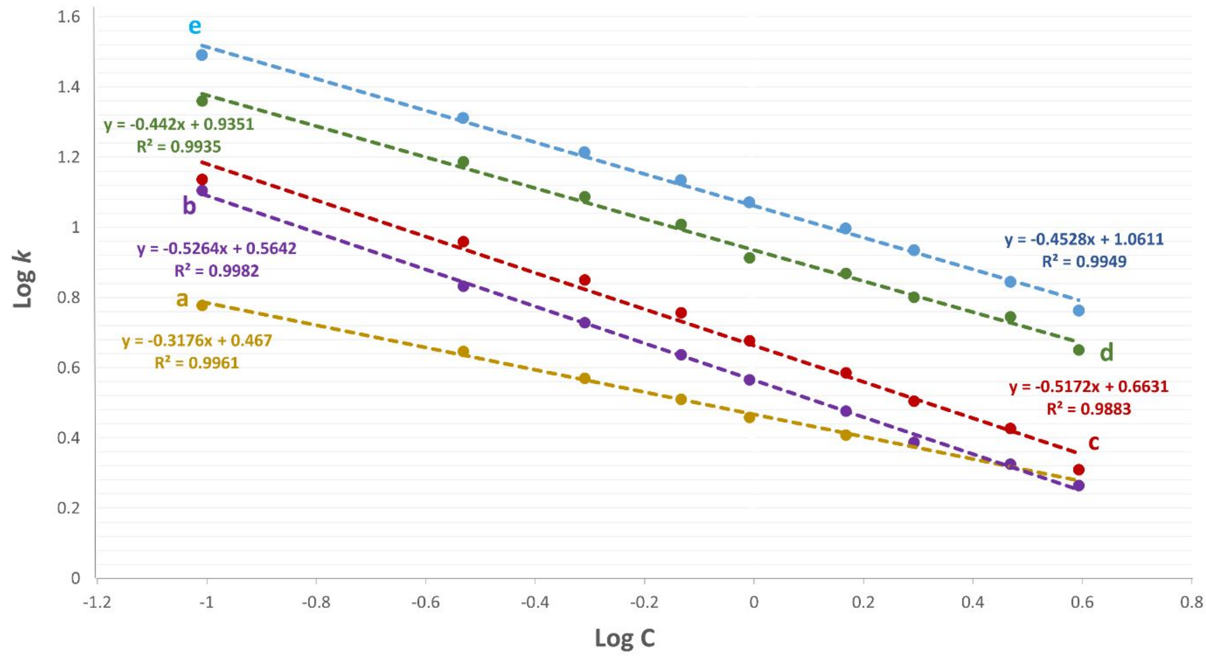

Fig. S1 The relationship between the concentration of perfluoroheptanoic acid in the mobile phase (expressed as Log C (in mM)) and the retention of the studied compounds (expressed as Log k (retention factor). (a) nicosulfuron; (b) metsulfuron: (c) chlorsulfuron; (d) tribenuron methyl; (e) sulfosulfuron.

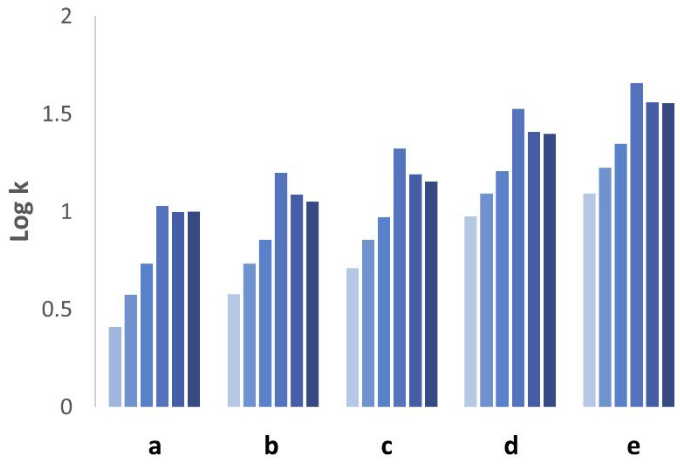

Fig. S2 The effect of salt concentration on the elution of the target compounds by ion-repulsion. Increased color intensity indicates increased ammonium acetate concentration (9, 18, 35, 70, 105, and 140 mM). Increased salt concentration mitigates ion-repulsion by mitigating electrostatic interactions. (a) nicosulfuron; (b) metsulfuron: (c) chlorsulfuron; (d) tribenuron methyl; (e) sulfosulfuron.
